# Subliminal enhancement of predictive effects during syntactic processing in the left inferior frontal gyrus: an MEG study

**DOI:** 10.3389/fnsys.2014.00217

**Published:** 2014-11-03

**Authors:** Kazuki Iijima, Kuniyoshi L. Sakai

**Affiliations:** ^1^Department of Basic Science, Graduate School of Arts and Sciences, The University of TokyoMeguro-ku, Japan; ^2^CREST, Japan Science and Technology AgencyChiyoda-ku, Japan; ^3^Japan Society for the Promotion of ScienceChiyoda-ku, Japan

**Keywords:** MEG, sentence processing, syntax, frontal cortex, prediction, consciousness

## Abstract

Predictive syntactic processing plays an essential role in language comprehension. In our previous study using Japanese object-verb (OV) sentences, we showed that the left inferior frontal gyrus (IFG) responses to a verb increased at 120–140 ms after the verb onset, indicating predictive effects caused by a preceding object. To further elucidate the automaticity of the predictive effects in the present magnetoencephalography study, we examined whether a *subliminally* presented verb (“subliminal verb”) enhanced the predictive effects on the sentence-final verb (“target verb”) unconsciously, i.e., without awareness. By presenting a subliminal verb after the object, enhanced predictive effects on the target verb would be detected in the OV sentences when the transitivity of the target verb matched with that of the subliminal verb (“congruent condition”), because the subliminal verb just after the object could determine the grammaticality of the sentence. For the OV sentences under the congruent condition, we observed significantly increased left IFG responses at 140–160 ms after the target verb onset. In contrast, responses in the precuneus and midcingulate cortex (MCC) were significantly *reduced* for the OV sentences under the congruent condition at 110–140 and 280–300 ms, respectively. By using partial Granger causality analyses for the OV sentences under the congruent condition, we revealed a bidirectional interaction between the left IFG and MCC at 60–160 ms, as well as a significant influence from the MCC to the precuneus. These results indicate that a top-down influence from the left IFG to the MCC, and then to the precuneus, is critical in syntactic decisions, whereas the MCC shares its task-set information with the left IFG to achieve automatic and predictive processes of syntax.

## Introduction

Human language consists of more than linear strings of words: hierarchical syntactic structures of a sentence are constructed by recursively merging a pair of syntactic objects (Chomsky, 1995). Understanding the processes by which syntactic structures are constructed is thus crucial for elucidating the neural mechanisms underlying the human language faculty. Recently, computational parsing theories with incremental predictions based on syntactic structures have been developed (Levy, 2008; Hale, 2011). According to these theories, the difficulty of processing a given phrase can be quantitatively explained by deviations from a prediction about the syntactic features of upcoming words in a sentence; such a prediction is based on the incrementally constructed syntactic structures. Assuming that a preceding noun phrase (NP) with a case marker (dative or accusative) in a Japanese sentence provides information about the argument structures of a sentence-final verb, we have shown that predictable canonical sentences increased the left inferior frontal gyrus (IFG) responses to the verb in our recent magnetoencephalography (MEG) study (Inubushi et al., 2012). In another MEG study with visually presented object-verb (OV) sentences, we showed that the left IFG responses to a verb significantly increased in a syntactic decision task, at 120–140 ms after the verb onset (Iijima et al., 2009). We interpreted this component as “predictive effects” caused by a preceding object with an accusative case marker (Acc, “-*o*”), such that a transitive verb (vt) was the only grammatical verb type for the final verb. Subject-verb (SV) sentences may lack such strong predictive effects, because the NP with a nominative case marker (Nom, “-*ga*”) cannot fully specify the following verb types, such as an intransitive verb (vi), vt, and copular verb (“*desu*, *da*, etc.,” which are similar to “*be*, etc.” in English) associated with a nominal/adjectival predicate. In that previous study (Iijima et al., 2009), we observed such predictive effects only for OV sentences in the syntactic decision task compared with other tasks (e.g., a semantic decision task). By presenting a verb *subliminally* (“subliminal verb” hereafter), enhanced predictive effects on the sentence-final verb (“target verb” hereafter) in OV sentences would be detected even in a single task of syntactic decision. Moreover, such enhancement is expected when the *transitivity* (vt or vi) of the target verb matched with that of the subliminal verb, because the subliminal verb just after the object could determine the grammaticality of the sentence.

**Figure 1:**
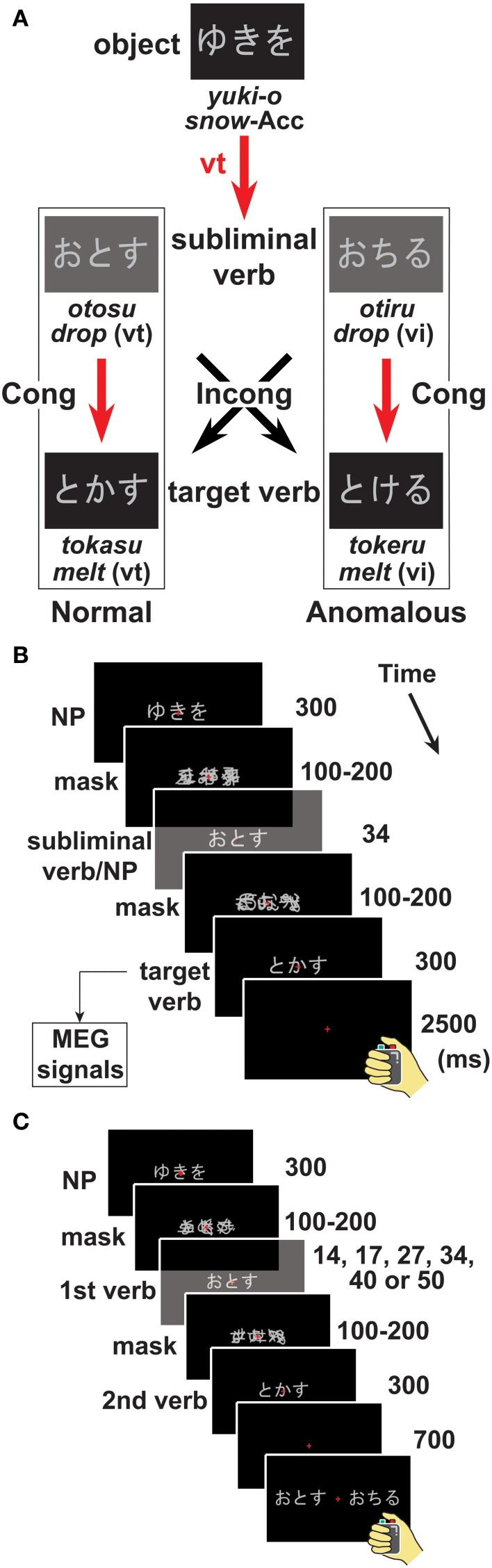
**A paradigm with subliminal stimuli**. We presented two-word sentences such as an object-verb (OV) sentence [e.g., “*yuki-o tokasu*”: “(*someone*) *melts snow*”] and a subject-verb (SV) sentence (e.g., “*yuki-ga tokeru*”: “*snow melts*”). The transitive verb (vt) and intransitive verb (vi) were both morphologically and semantically related (see Table 1), but always *different* words, just as in the “*raise*/*rise*” distinction in English. **(A)** Examples of visually presented stimuli of an OV sentence. In a syntactic decision task, participants decided whether a presented sentence was syntactically normal or anomalous. A *supraliminally* presented verb (“target verb”) appeared at the end of each trial for the participants to respond to. A *subliminally* presented verb (“subliminal verb”) was inserted between a noun phrase (NP) and the target verb. The target verb was either congruent (Cong) or incongruent (Incong) with the subliminal verb in terms of their verb transitivity (vt or vi). Red arrows indicate a *prediction* about the verb, provided by an object with an accusative case marker (Acc), such that the following vt is normal, and the following vi is anomalous. **(B)** A single trial in the syntactic decision task. We sequentially presented an NP, a subliminal verb or NP, and a target verb, together with a forward mask and a backward mask before and after the subliminal verb, respectively. We focused on cortical responses to target verbs, and we presented the masks with random intervals between 100 and 200 ms, so that cortical responses to target verbs were not confounded with those to the other stimuli. **(C)** A single trial in a forced-choice recognition task to assess the visibility of a masked first verb. At the end of this task, two stimuli were presented, and participants simply chose which stimulus had actually appeared as the first verb (interval, 14–50 ms). We made the stimulus presentation of each trial identical to that in the syntactic decision task, except that two verbs were presented as a choice stimulus.

We further hypothesize that the predictive effects caused by the preceding object actually represent early syntactic processes of determining the transitivity of the final verb. To further elucidate the automaticity of the predictive effects, we examined whether a subliminal verb after the object enhanced the predictive effects on the target verb unconsciously, i.e., without awareness (Figure 1A). We presented a subliminal verb for a limited time between two masks after the NP (Figure 1B). Participants were not notified of even the existence of subliminal stimuli during the experiment, although they were fully aware that a target verb would appear after the NP while paying attention to a sequence of stimuli. Therefore, they could not have expected to receive any information about the upcoming verb from the subliminal verb. As shown in Figure 1A, the target verb was either congruent (Cong) or incongruent (Incong) with the subliminal verb in terms of their transitivity, leading to four stimulus conditions: OV-Cong, SV-Cong, OV-Incong, and SV-Incong. The lexico-semantic relationships between the noun and subliminal verb, as well as between the noun and target verb, were always normal and equivalent among these four conditions. This strict semantic control is one of the merits of the present study.

We defined syntactically “normal OV” and “normal SV” sentences as object-vt and subject-vi combinations, respectively. From each of the normal OV and SV sentences, we made a syntactically anomalous sentence by simply exchanging the verb with the rest of a verb pair, which consisted of a morphologically and semantically related vt and vi (Table 1). Here we defined “anomalous OV” and “anomalous SV” sentences as those with an object (vi with “-*o*”) and subject (vt with “-*ga*”), respectively. From a normal OV sentence [e.g., “*yuki*-*o tok*-***as***-***u***(= vt)”: “(*someone*) *melts snow*”], we made an anomalous OV sentence [e.g., “*yuki*-*o tok*-***e***-***ru*** (=vi)”], which is ungrammatical, since a vi cannot take an object. From a normal SV sentence [e.g., “*yuki*-*ga tok*-***e***-***ru*** (=vi)”: “*snow melts*”], we made an anomalous SV sentence [e.g., “*yuki*-*ga tok*-***as***-***u*** (= vt)”], which is ungrammatical, since its error can be immediately corrected by the grammatical counterpart: either “*yuki*-*ga tok*-***e***-***ru***” (the verb type counterpart) or “*yuki*-***o*** *tok*-*as*-*u*” (the case marker counterpart) in this example. By presenting both normal OV and normal SV sentences, a judgment on the grammaticality would surpass a judgment on selectional restrictions, if any.

**Table 1:** **A list of 72 normal sentences**.

**Verb subgroup**	**Object-Verb (OV) sentence**		**Subject-Verb (SV) sentence**		**Translation of SV sentence**
	**Noun-Acc**	**vt**	**Noun-Nom**	**vi**	
I	*tama-o*	*at-e-ru*	*tama-ga*	*at-ar-u*	*the bullet hits* (*someone*)
II		*sor-as-u*		*sor-e-ru*	*the bullet misses*
I	*huku-o*	*kim-e-ru*	*huku-ga*	*kim-ar-u*	*clothes get selected*
II		*nur-as-u*		*nur-e-ru*	*clothes get wet*
I	*shiru-o*	*maz-e-ru*	*shiru-ga*	*maz-ar-u*	*sauce mixes*
II		*tar-as-u*		*tar-e-ru*	*sauce drips off*
I	*nuno-o*	*som-e-ru*	*nuno-ga*	*som-ar-u*	*the cloth gets dyed*
II		*moy-as-u*		*mo*(*y*)*-e-ru*	*the cloth gets burnt*
I	*oyu-o*	*tam-e-ru*	*oyu*-*ga*	*tam-ar-u*	*hot water pools*
II		*hiy-as-u*		*hi-e-ru*	*hot water cools*
I	*iki-o*	*tom-e-ru*	*iki-ga*	*tom-ar-u*	*the breath ceases*
II		*mor-as-u*		*mor-e-ru*	*the breath gets out*
I	*ine-o*	*u*(*w*)*-e-ru*	*ine-ga*	*uw-ar-u*	*the rice is planted*
II		*kar-as-u*		*kar-e-ru*	*the rice withers*
II	*kabe-o*	*kog-as-u*	*kabe-ga*	*kog-e-ru*	*the wall gets burnt*
III		*nao-s-u*		*nao-r-u*	*the wall gets fixed*
II	*kome-o*	*mur-as-u*	*kome-ga*	*mur-e-ru*	*the rice gets steamed*
III		*nok-os-u*		*nok-or-u*	*the rice remains*
II	*netsu-o*	*sam-as-u*	*netsu-ga*	*sam-e-ru*	*the fever wanes*
III		*kom-e-ru*		*kom-or-u*	*the fever pervades*
II	*yuki-o*	*tok-as-u*	*yuki-ga*	*tok-e-ru*	*snow melts*
III		*ot-os-u*		*ot-i-ru*	*snow drops*
II	*mado-o*	*yur-as-u*	*mado-ga*	*yur-e-ru*	*the window shakes*
III		*mi-se-ru*		*mi-e-ru*	*the window can be seen*
III	*ashi-o*	*hit-as-u*	*ashi-ga*	*hit-ar-u*	*the legs soak*
I		*mag-e-ru*		*mag-ar-u*	*the legs bend*
III	*waza-o*	*ik-as-u*	*waza-ga*	*ik-i-ru*	*techniques get utilized*
I		*kak-e-ru*		*kak-ar-u*	*techniques succeed*
III	*huta-o*	*maw-as-u*	*huta-ga*	*maw-ar-u*	*the lid gets screwed*
I		*shim-e-ru*		*shim-ar-u*	*the lid gets closed*
III	*mizu-o*	*mit-as-u*	*mizu-ga*	*mit-i-ru*	*water brims in* (*something*)
I		*tam-e-ru*		*tam-ar-u*	*water pools*
III	*tabi-o*	*nob-as-u*	*tabi-ga*	*nob-i-ru*	*the travel gets extended*
I		*o*(*w*)*-e-ru*		*ow-ar-u*	*the travel ends*
III	*boya-o*	*ok-os-u*	*boya-ga*	*ok-i-ru*	*small fire occurs*
I		*tom-e-ru*		*tom-ar-u*	*small fire stops*

In every two rows with the same noun, two pairs of a transitive verb (vt) and an intransitive verb (vi) are shown, where each pair in a row is morphologically related and shares the same meanings. For a single trial, a subliminal verb and a target verb were chosen from each of the two vt-vi pairs (see Figure 1A). According to Shibatani (1990), verb pairs of vt and vi can be divided into three verb subgroups in terms of their morphological/phonological regularity: I (vt/vi: -e-ru/-ar-u), II (-as-u/-e-ru), and III (others). Verbs from two different subgroups were selected for each noun.

A recent MEG study has shown that phonologically predictable words (e.g., spoken sounds from written words) increased the left IFG responses at around 150 ms after the word onset (Sohoglu et al., 2012), while semantically or syntactically *unpredictable* words increased neural responses in *posterior* regions (DeLong et al., 2005; Dikker et al., 2009). Moreover, previous neuroimaging studies have reported an *increase* of the left IFG responses reflecting syntactic predictive effects (Iijima et al., 2009; Inubushi et al., 2012; Santi and Grodzinsky, 2012). These previous studies have indicated that syntactic prediction generated by a preceding NP remains effective for as long as 300–900 ms. We thus expect to observe the increased left IFG responses within this time frame under the OV-Cong condition (Figure 1B). Our focus is neither on simple priming effects of transitivity (i.e., congruency) nor on the generation of a prediction itself (i.e., NP-type effects), but on the *enhancement* of predictive effects due to a subliminal verb. We infer that the left IFG shows enhanced responses under the OV-Cong condition when compared with the combined conditions of *SV-Cong* and *OV-Incong*, i.e., (SV-Cong + OV-Incong), in which the congruency and NP-type effects are separately controlled. We assume that the OV-Incong condition is ineffective and neutral regarding the enhancement; the use of this condition is thus a better control than that of the OV condition without a subliminal verb, because the stimulus presentation (including the presence of a subliminal verb) is physically controlled among the compared conditions. Moreover, our prediction is more focused than an interaction of sentence structure by congruency, i.e., (OV-Cong + SV-Incong) vs. (SV-Cong + OV-Incong), because we expect no enhancement under the SV-Incong condition. To examine whether the left IFG responses were robust enough across both spatial and temporal domains, we applied whole-brain analyses of MEG responses in an unbiased manner, where neither particular regions nor temporal bins were selected *a priori*, which was equivalent to performing all possible functional region of interest (ROI) analyses (Friston and Henson, 2006).

In an SV sentence, the verb type cannot be uniquely specified, and thus the bottom-up determination of the transitivity from a presented stimulus had to be done for both subliminal and target verbs. This possible interference would lead to longer reaction times (RTs) for the SV sentences than for the OV sentences, irrespective of the Cong and Incong conditions. As a control for the interference from a subliminal verb, we compared behavioral data for the SV and OV sentences when a subliminal NP was presented instead of a subliminal verb. Because a subliminal NP was always the same as the preceding NP in each trial, the use of subliminal NPs introduced no confounding effects.

Previous functional magnetic resonance imaging (fMRI) studies of normal participants have established that the left IFG and the left lateral premotor cortex play a crucial role in syntactic processes (Stromswold et al., 1996; Dapretto and Bookheimer, 1999; Embick et al., 2000; Hashimoto and Sakai, 2002; Friederici et al., 2003; Musso et al., 2003; Suzuki and Sakai, 2003; Kinno et al., 2008); these regions have been proposed as putative grammar centers (Sakai, 2005). Moreover, our recent fMRI study has shown that the left IFG responses were parametrically modulated by “the Degree of Merger (DoM),” which was defined as the maximum depth of merged subtrees (i.e., Mergers) within an entire sentence (Ohta et al., 2013). “Merge” is a simple local structure-building operation proposed by modern linguistics; Merge would be theoretically “costless,” requiring no driving force for its application (Saito and Fukui, 1998; Chomsky, 2004; Fukui, 2011). We suggest that structure-building involves automatic Merge processes, which would be facilitated by syntactic prediction from a preceding phrase. It has been suggested that a simple type of information integration is facilitated without awareness (Mudrik et al., 2014). There has been recent supporting evidence that sentence processing actually occurs in the absence of awareness (Batterink and Neville, 2013; Axelrod et al., 2014), while some subliminal priming studies have targeted lower levels of phonology, morphology, and lexico-semantics (Kouider and Dehaene, 2007; Nakamura et al., 2007; Lehtonen et al., 2011; Wilson et al., 2011). We hypothesize that further subliminal processes at the lexical level extend to hierarchically higher syntactic processes without awareness; the subliminal syntactic process is a critical assumption in this hypothesis.

Another candidate region for response modulation under the OV-Cong condition is the midcingulate cortex (MCC), which is involved in task-set formation (Dosenbach et al., 2006; Hyafil et al., 2009). We also tried to elucidate causal influences among these regions by using partial Granger causality analyses (Guo et al., 2008; Barrett et al., 2010). Under the OV-Cong condition, we expect that causal interactions between the left IFG and other regions were enhanced. Our present study should help to clarify the neural basis of syntactic processes that are both automatic and predictive.

## Materials and methods

### Participants

The participants in the MEG experiments were 16 native Japanese speakers. One participant, who reported that he was able to detect the subliminal verbs during the MEG experiment, was excluded from the behavioral and MEG data analyses, leaving a total of 15 participants (19–43 years; four females). All of them showed right-handedness (laterality quotients: 87–100) as determined by the Edinburgh inventory (Oldfield, 1971). In the pilot study for determining an appropriate interval of subliminal stimuli, 10 other native Japanese speakers (22–35 years; one female) participated. All participants were neurologically normal without any psychiatric symptoms. Written informed consent was obtained from each participant after the nature and possible consequences of the studies were explained. Approval for these experiments was obtained from the institutional review board of the University of Tokyo, Komaba.

### Stimuli

In most languages, there are two types of intransitive verbs: unaccusative verbs and unergative verbs. The subjects of unaccusative verbs, as well as the objects of transitive verbs, have the semantic role of “theme” (the entity undergoing the effect of some action). In this study, in order to equate semantic factors among the conditions, we used unaccusative verbs alone for the intransitive verbs, so that the NPs of both OV and SV sentences had the same semantic role. Moreover, we used the same set of nouns for both sentence structures. Note that in Japanese a null nominative-case pronoun is allowed as a subject, as well as in Spanish and Italian, and we omitted from the SOV sentences a subject whose semantic role is “agent” (the entity instigating some action). The following examples clarify the distinction between SVO and SV sentences in English, which is similar to the OV and SV distinction:

- The coach (= agent) substituted (= vt) John (= theme) for Dave, and I (= agent) would have done so,
- John (= theme) substituted (= vi) for Dave, and I (= theme) would have done so,

as “done so” substitutes for the verb phrase “substituted John for Dave” or “substituted for Dave.”

The distinction between vt and vi, i.e., verb transitivity, is one of the universal aspects of syntactic features among natural languages. In the Japanese language, there are a number of verb pairs, each of which consists of a morphologically and semantically related vt and vi (e.g., “*at*-*e*-*ru*” and “*at*-*ar*-*u*”; Table 1). The vt-vi pair relationships are determined by complex rules of morphosyntax (Shibatani, 1990), similar to the distinction of “*raise*/*rise, fell/fall, lay/lie, set/sit*” in English. There are some Japanese verbs which lack such morphological distinction [e.g., “*hirak*-*u*” (“*open*”) for both vt and vi], but we did not use them in the present study. Two vt-vi pairs were chosen for each noun, which was always inanimate and semantically related with the four verbs. For every trial, a subliminal verb and a target verb were chosen from each of the two vt-vi pairs (e.g., “*at*-*e*-*ru*” and “*sor*-*as*-*u*”; see Table 1), so that the subliminal and target verbs had neither direct semantic nor morphological/phonological relationships that may have affected congruency.

**Figure 2:**
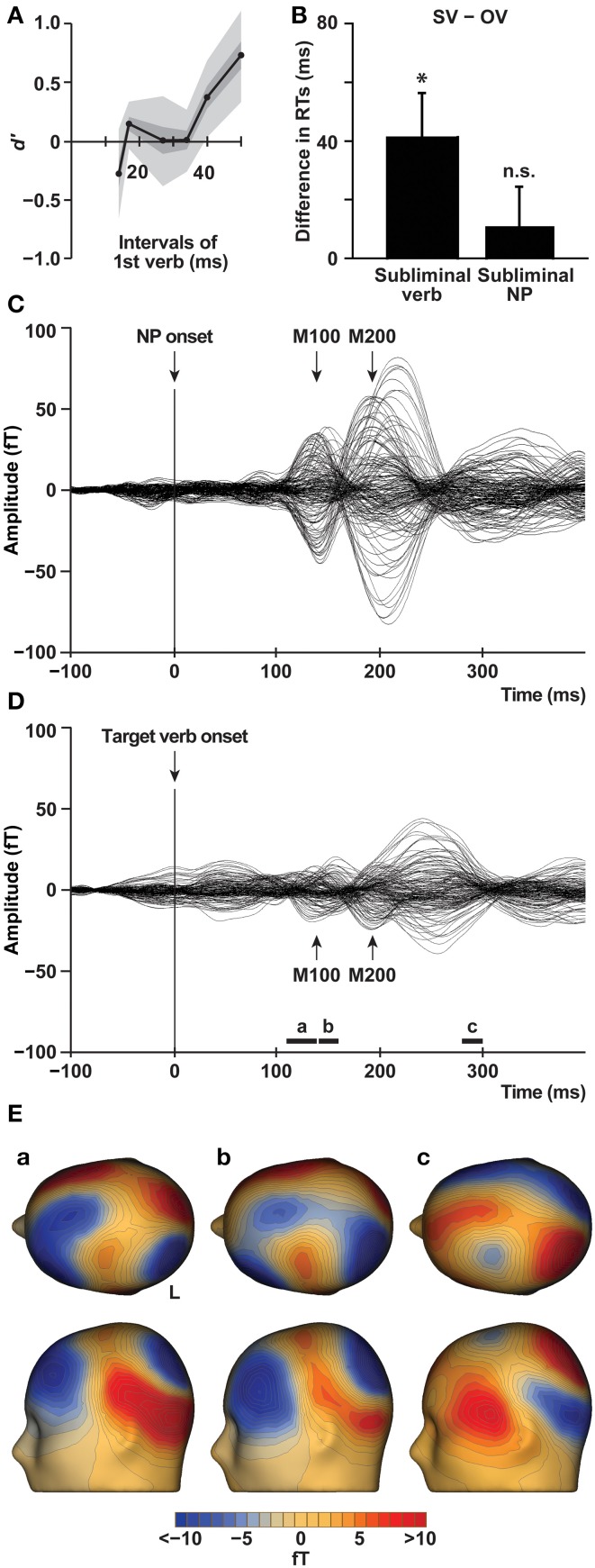
**Behavioral results and MEG signals**. **(A)** Results of the forced-choice recognition task. The discriminability of stimuli (*d*′) is shown against various intervals of the first verb. The SEMs and 95% confidence intervals (Bonferroni-corrected) are shown in the lighter and lightest shades, respectively (*n* = 10). The results showed that subliminal verbs of 34 ms were too short to be seen. **(B)** Interference from a subliminal verb for the SV sentences in the syntactic decision task. The histograms show the differences in RTs obtained by subtracting RTs for the OV sentences from those for the SV sentences (mean ± s.e.m., *n* = 15), averaged under both Cong and Incong conditions. A significantly increased difference in RTs was observed for a subliminal verb, but not for a subliminal NP. An asterisk denotes a significant difference (*P* = 0.05, paired *t*-test). **(C)** MEG signals for the NPs from artifact-free and correct trials, averaged for all of the four conditions and across 15 participants, are shown for each sensor before normalization. **(D)** MEG signals for the target verbs are shown for each sensor. The black bars (a: 110–140; b: 140–160; c: 280–300 ms) denote all of the temporal bins that showed any significant differences between the tested conditions (Figures 3A–C). **(E)** MEG topographies on the scalp averaged under the OV-Cong condition, at each temporal bin of a-c **(D)**. The upper and lateral scalp surfaces are shown.

Each word stimulus was either an NP (a noun and a case marker) or verb (Figure 1B), and always consisted of three letters (three moras or syllables) spelled only in kana (Japanese phonograms) to ensure a consistent reading time. In each trial starting from an NP for 300 ms, a mask was presented with a random interval of 100, 117, 134, 150, 167, 184, or 200 ms. This mask served as a *forward* mask for the next-coming subliminal verb, which was presented for 34 ms. A *backward* mask followed this subliminal stimulus with the same random intervals. A target verb was then presented for 300 ms. By randomizing the intervals of backward and forward masks, we separated the effects on the target verb from any responses to an NP, subliminal verb, or mask stimuli (Figure 1B). This procedure enabled us to minimize the interference of preceding stimuli with the baseline activity of the target verbs. Moreover, overlapping between responses to the backward mask and initial responses to the target verb could not explain any response differences among the conditions, because the mask stimulus presentation was common across all tested conditions. We also confirmed that the larger baseline noises (see Figure 2D) were restricted to posterior sensors. The inter-trial interval was randomly varied within the range of 5 ± 0.5 s to reduce any periodical noises.

Mask stimuli, which should be unreadable while retaining some features of the kana stimuli, were made in the following procedures. Three verb stimuli were selected, and for each stimulus three kana letters were rotated randomly at three different angles (±90°, 180°). We made 27 different mask stimuli (see Figure 1B) by superimposing one of these three resultant stimuli with two new stimuli consisting of three pseudoletters. In a pilot study in which each mask stimulus were presented alone for 200 ms, we tested whether any of the “letters” could be identified as any kana letter. In the 243 trials tested, only two answers matched with the original letters, and this result was not significantly different from chance (*P* > 0.6, *t*-test).

We prepared 36 verb pairs of vt and vi, and made 72 normal sentences (Table 1), each of which consisted of an NP and one of these verbs as a target verb (36 each for OV and SV sentences). Using *Google* (http://www.google.co.jp/), we calculated a transitional probability from an NP to a verb within a sentence, and there was no significant difference between the normal OV and SV sentences [*T*_(35)_ = −0.053, *P* > 0.9 (paired *t*-test)]. We made 72 anomalous sentences from these normal sentences, exchanging the vt and vi for the corresponding NPs (36 each for the OV and SV sentences). For each of the normal and anomalous sentences, we tested two different subliminal verbs, corresponding to either the Cong or Incong condition (see Figure 1A). For each of four conditions (i.e., OV-Cong, SV-Cong, OV-Incong, and SV-Incong), there were thus 72 combinations for the set of an NP, a subliminal verb, and a target verb. In addition, we prepared 144 possible combinations for the set of an NP, a *subliminal NP* (34 ms), and a target verb (72 each for normal and anomalous sentences); we randomly chose 72 combinations (on average, 36 each for OV and SV sentences) for each participant. Each of these different combinations with subliminal stimuli (verb or NP) was tested only once for each participant.

Stimulus presentation and behavioral data collection were controlled using Presentation software (Neurobehavioral Systems Inc., Albany, CA) and an NI-DAQ interface board (National Instruments, Austin, TX). Visual stimuli in gray against a dark background were projected with a refresh rate of 60 Hz (i.e., 16.67 ms for one video frame) from outside of the shield room onto the translucent screen within a visual angle of 5.7°, using a Digital Light Processing projector (TDP-EX20J; Toshiba, Tokyo, Japan) equipped with a projection lens (modified by NewOpto, Tokyo, Japan). For fixation to minimize eye movements, a red cross was always shown at the center of the screen, and the participants were instructed to stare at it and refrain from blinking before the response.

### Tasks

Native Japanese speakers judged the grammaticality of two-word sentences, i.e., an NP with a case marker and a target verb (Figure 1B). The participants were instructed to respond to the target verb by pressing one of two buttons (right or left) as quickly as possible by using the right hand alone. Assignments of the two buttons for the judgment of sentences as normal or anomalous were counterbalanced across participants. This syntactic decision task, *per se*, was designed in the same way as in our previous study (Iijima et al., 2009). The syntactic decision task could not be solved on the basis of the lexico-semantic relationship between a noun and a target verb, as it was always correct as explained above.

Each of the four MEG runs performed on a single day for any given participant included 90 trials mixed randomly with subliminal verb and subliminal NP stimuli. Each of the four conditions (i.e., OV-Cong, SV-Cong, OV-Incong, and SV-Incong) consisted of 72 trials for each of the 15 participants, resulting in 1080 observations per condition for an entire experiment. For all participants, the orders of sentence structures (OV or SV), congruency, and grammaticality were fully randomized and counterbalanced. Only trials with participants' correct responses were used for analyzing RTs and MEG data.

### Pilot study for determining an appropriate interval of subliminal stimuli

In order to test whether the participants were actually unaware of a subliminal verb for 34 ms, we performed another pilot study with a forced-choice recognition task, thereby varying the interval of a masked verb (first verb) (Figure 1C). We made the stimulus presentation of each trial identical to that in the syntactic decision task, using the same set of 288 combinations for the set of an NP, a masked first verb, and a second verb, except that two verbs were presented as a choice stimulus, which remained on the screen until the participant responded. In each trial, participants chose which of the two verbs had actually appeared as the first verb, simply neglecting the NP and second verb. The participants were explicitly informed of the presence of a first verb even when it was too short to recognize. For each choice stimulus, a distractor was taken from the particular vt-vi pair of the first verb (Table 1). There were two runs, in which we used a fixed refresh rate of the Digital Light Processing projector (one with 60 Hz, and the other with 75 Hz). For the refresh rate at 60 Hz (i.e., 16.67 ms for one video frame), we randomly tested three intervals of the first verb (17, 34, or 50 ms set with the Presentation software); for the refresh rate at 75 Hz (i.e., 13.33 ms for one video frame), we also randomly tested three intervals of the first verb (14, 27, or 40 ms). We calculated *d*′, i.e., the discriminability of stimuli, from each participant's hit and false-alarm rates.

### MEG and MRI data acquisition

The MEG data were acquired with a 160-channel whole-head system (MEGvision; Yokogawa Electric Corporation, Kanazawa-city, Japan), and they were digitized with an on-line bandwidth of 0.3–1000 Hz and a sampling rate of 2000 Hz. This bandwidth was set according to the Nyquist sampling theorem. At the time of setting up the MEG system, there was no salient noise just below 2000 Hz that might have caused aliasing in our target frequency of 2–30 Hz. We basically followed the same procedures described in our previous studies (Iijima et al., 2009; Inubushi et al., 2012). The MEG signals within the period of −100 to +400 ms from the target verb onset were analyzed using the BESA software, version 5.2 (BESA, Gräfelfing, Germany). Under each condition for a single participant, only artifact-free trials (peak-to-peak amplitude <2500 fT) with correct responses were averaged without filtering. The signals from −100 to 0 ms at the target verb onset were used as a baseline, which was within the period of presenting the backward mask (see Figure 1B). The baseline-corrected MEG signals were then band-pass filtered from 2 to 30 Hz to eliminate large eye movement noises, which may shift the baseline level from zero. While this band-pass filtering removed information of the gamma band (above 30 Hz), some recent studies revealed the important role of the beta band (13–30 Hz) in language processing (Weiss and Mueller, 2012). Artifact-free trials with participants' correct responses accounted for approximately 85% of observations, and this percentage did not differ significantly across the different conditions (*P* > 0.9).

For mapping with the individual brain, high resolution T1-weighted MR images (repetition time, 8.4 ms; echo time, 2.6 ms; flip angle, 25°; field of view, 256 × 256 mm^2^; resolution, 1 × 1 × 1 mm^3^) were acquired using a 3.0-T Scanner (Signa HDxt; GE Healthcare, Milwaukee, WI). The sensor positions for each of four runs were realigned with five fiducial markers (small coils) on the head surface, and coregistered with a least-squares fit algorithm to the MR images (MEG Laboratory; Yokogawa Electric Corporation, Kanazawa-city, Japan); we attached MR markers (alfacalcidol beads; diameter: 3 mm) at the same positions as the fiducial markers. Using BrainVoyager QX 1.8 software (Brain Innovation, Maastricht, Netherlands), each individual brain was normalized to the image of the Montreal Neurological Institute standard brain, which was already transformed into the Talairach space (Talairach and Tournoux, 1988). The gray and white matter of the transformed standard brain was segmented, and their boundary was then partitioned into 3445 cortical patches with a mean distance of 5.6 mm (Kriegeskorte and Goebel, 2001). We confirmed that the cortical patches were appropriately created in both the lateral and medial regions. Using the transformation matrix for normalization, the cortical patches on the standard brain were inversely transformed into each participant's space, and were used for cortex-based data analyses.

### MEG data analyses

An overview of the MEG data analyses is as follows. We first estimated current dipoles in each participant's space. For each cortical patch's current density in a temporal bin, we compared cortical currents between specified conditions across the participants (*n* = 15). For the clusters, each of which was the group of selectively responsive patches, we used a cluster permutation test to calculate each cluster's corrected *P*-value across the whole brain. We further corrected each cluster's *P*-value across temporal bins by using the false discovery rate. Through these two steps, we corrected the *P*-values across both the spatial and temporal domains (*P*_corr_ = 0.05); a similar correction method in temporal and then spatial domains was adopted in a previous MEG study (Brennan and Pylkkänen, 2012). The details of our procedures are as follows.

Using the minimum norm estimates of currents computed with BESA 5.2, we modeled the distribution of cortical activation underlying the MEG signals, which were averaged among all correct trials under each condition. A current dipole was perpendicularly placed at each center of the 3445 transformed cortical patches; the multiple dipoles approximated any spatial distributions of currents on the cortex, without assuming the number or positions of responsive dipoles (Dale and Sereno, 1993; Hämäläinen et al., 1993). Using in-house programs on MATLAB (http://www.mathworks.com/products/matlab), the current density at each cortical patch was obtained by dividing the strength of each current dipole by the mean area of the cortical patches. The current density at each cortical patch was averaged for a bin of 20 ms; the temporal bin was slid in 10 ms steps over the 100–400 ms period after the target verb onset, resulting in 29 temporal bins. We have adopted the same procedures for temporal bins in our previous studies (Iijima et al., 2009; Inubushi et al., 2012).

We first reduced the search spaces by selecting potentially responsive cortical patches, in which the current density averaged across temporal bins of 0–400 ms was larger than the mean baseline responses (−100–0 ms) under all of the four conditions (paired *t*-tests among the participants; uncorrected *P* < 0.001). For each cortical patch's current density in a temporal bin, we then compared cortical currents between specified conditions across the participants (paired *t*-tests). We chose responsive patches whose absolute *t*-values were larger than the threshold of *T*_(14)_ = 3.8 (selection criteria: *Z* = 3.3; uncorrected *P* = 0.001). If the distance between two of those patches in the Talairach space was within 7 mm, we paired them and connected the adjacent pairs of patches as a *cluster*. An isolated responsive patch was also regarded as a cluster. Within each cluster, the *t*-values (absolute values) from responsive patches were summed up to represent the cluster. The statistical significance of observing those clusters was then evaluated and corrected for multiple comparisons across the whole brain by using the following cluster permutation test (Maris and Oostenveld, 2007). For all cortical patches of the brain, the current density was exchanged between specified conditions in some of the 15 participants, and the *t*-values were recalculated, followed by the generation of new clusters. The largest sum of the *t*-values was then determined among the imaginary clusters for each permutation. There were 2^15^ = 32,768 permutations, which produced a reference distribution of the sum of *t*-values for determining the corrected *P*-values of observed clusters. Next, each cluster's *P*-value was further corrected for multiple comparisons across temporal bins by using the false discovery rate based on the Benjamini-Hochberg procedure (Benjamini and Hochberg, 1995). To visualize the resulting significant cluster, color spheres (7 mm in diameter) were placed on cortical patches. Using SPM8 (http://www.fil.ion.ucl.ac.uk/spm/software/spm8) on MATLAB, these spheres were spatially filtered with a Gaussian (full width at half maximum, 7 mm) and superimposed onto the Talairach-transformed standard brain with MRIcron (http://www.cabiatl.com/mricro/mricron/index.html).

### Partial granger causality analyses

By using Granger causality analyses (Granger, 1969; Geweke, 1982), we further examined which pairs of two clusters had significant causality for a specified period. Among the three clusters that we selected, there were six possible *causal influences*, e.g., from a cluster *X* to a cluster *Y*. According to the standard Granger causality, a variable *x* (a time series of the cluster *X*) “Granger-causes” a variable *y* (a time series of the cluster *Y*), if information in the past of *x* (with specified time-lags) helps predict the future of *y* with better accuracy than is possible when considering only information in the past of *y* itself. Partial Granger causality is a superior extension of the standard Granger causality, in that it takes into account causal influences of any exogenous inputs and latent variables (Guo et al., 2008; Barrett et al., 2010). This method is suitable for our present study, because it can adequately examine multiple clusters that may receive exogenous common inputs under all conditions. Under each condition, the time series data of the current density without binning were averaged within each cluster for every participant. For this averaging, we considered only the magnitude of the current density at each cortical patch, since the orientation of a dipole was fixed perpendicularly in a similar direction for adjacent patches in a cluster. Based on the results of cortical responses, the averaged time series data were divided into three periods of 100 ms relative to a reference time of 160 ms (determined by the left IFG responses): 60–160, 160–260, and 260–360 ms. Such 100-ms periods have been used in Granger causality analyses of cortico-cortical interactions in various human systems (Ploner et al., 2009; Lou et al., 2011).

Using a MATLAB Toolbox called GCCA (Granger Causality Connectivity Analysis) (Seth, 2010), we removed the linear trends from the time series data with the function cca_detrend. With the function cca_rm_ensemblemean, non-stationarities during a single period were further removed by subtracting the ensemble mean across participants, and each participant's standard deviation was divided by the ensemble standard deviation. The non-stationarities of the resultant data were not statistically significant (*P* > 0.05) according to the previously proposed test (Kwiatkowski et al., 1992), implemented as the function cca_kpss_mtrial.

Using the time series data of 15 participants regarded as 15 repetitions, partial Granger causalities for the six causal influences were calculated with the function cca_partialgc_doi_permute. A model order, i.e., the number of time-lags used in a multivariate autoregressive model, was specified by the function cca_find_model_order_mtrial, using Akaike information criterion (Akaike, 1974). The range of a model order was first set between 10 and 20 ms as used previously (Gow et al., 2008; Gaillard et al., 2009), and the resultant optimal model order was between 10 and 16.5 ms. This time range is consistent with the latency of cortico-cortical evoked potentials from the parietal regions to the frontal regions (Matsumoto et al., 2012). Any spatial spread of the MEG field might produce spurious causal influences among multiple regions. Based on simulated data, it has been recommended that causality analyses be performed on estimated cortical currents, but not on signals of MEG sensors, while contrasting specified conditions to cancel out the general effects of field spread (Schoffelen and Gross, 2009; Gross et al., 2013). Following this recommendation, we examined the differences in causalities between specified conditions. The statistical significance of the observed differences was evaluated by using the following permutation test for each condition. The time series data were divided into bins of 20 ms, which should be longer than the optimal model order, and these bins from multiple participants were permutated randomly and independently for each cluster. For each pair of *i*-th permutations (*i* = 1, 2, …, 1000) for specified conditions, a *difference* in partial Granger causalities was recalculated to produce a reference distribution for determining *P*-values of observed differences. In (SV-Cong + OV-Incong), we calculated partial Granger causalities separately for the SV-Cong and OV-Incong conditions, and averaged the results. These *P*-values were further corrected for multiple comparisons across six causal influences using the false discovery rate based on the Benjamini-Hochberg procedure (*P*_corr_ = 0.05).

For each condition, the *P*-value of a partial Granger causality was also determined with a permutation test as explained above. Because the statistical thresholds of partial Granger causalities were different for different periods and conditions, we presented the partial Granger causality normalized with its own threshold. For each cluster pair with a significant influence, we further examined the difference of two directed influences, which was determined by the permutation test (Roebroeck et al., 2005).

## Results

### Assessment of the visibility of masked stimuli

In the pilot study with the forced-choice recognition task, we assessed the visibility of a first verb by varying the interval of this masked stimulus itself (Figure 1C). Among the intervals of 50, 40, 34, 27, 17, and 14 ms, the mean *d*′ data for the 50 and 40 ms intervals were significantly different from zero (Bonferroni-corrected) [50 ms: *d*′ (mean ± s.e.m.) = 0.78 ± 0.12, *T*_(9)_ = 6.4, *P*_corr_ = 0.0008; 40 ms: *d*′ = 0.40 ± 0.10, *T*_(9)_ = 4.0, *P*_corr_ = 0.02] (Figure 2A), indicating that the first verb was clearly visible to the participants. In contrast, the mean *d*′ data for the other intervals were not significantly different [34 ms: *d*′ = 0.020 ± 0.083, *T*_(9)_ = 0.24, *P*_corr_ > 0.9; 27 ms: *d*′ = 0.016 ± 0.12, *T*_(9)_ = 0.13, *P*_corr_ > 0.9; 17 ms: *d*′ = 0.16 ± 0.062, *T*_(9)_ = 2.6, *P*_corr_ = 0.2; 14 ms: *d*′ = −0.28 ± 0.12, *T*_(9)_ = 2.4, *P*_corr_ = 0.3]. For the MEG experiments, we thus chose the longest interval of 34 ms for subliminal stimuli (verb or NP) of which the participants were unaware, so that the presence of a subliminal verb was long enough to affect syntactic decisions.

In order to confirm that the participants in the MEG experiments were indeed unaware of the subliminal verbs, two additional examinations were performed *after* the MEG recordings. First, the participants were notified for the first time that a subliminal verb actually appeared between an NP and a target verb, and asked if they were aware of any subliminal verbs or not. Only one participant reported that he was aware of the existence of subliminal verbs during the MEG experiment; this participant was thus excluded from the behavioral and MEG data analyses. Secondly, we carefully assessed the visibility of the first verb by using a forced-choice recognition task with a fixed interval of 34 ms (100 trials for each participant). Consistent with the results of the pilot study, the mean *d*′ for the first verb was not significantly different from zero [*d*′ = 0.20 ± 0.12, *T*_(14)_ = 1.6, *P* = 0.1]. These results confirmed that the participants remained unconscious to subliminal verbs even after repeated exposures to the stimuli during the MEG experiment.

### Behavioral results

The behavioral data of the syntactic decision task performed during the MEG experiments are shown in Table 2. From the trials with a subliminal NP, we analyzed behavioral data, but not MEG signals, as those trials were half of the trials with a subliminal verb. As regards the accuracy, there were neither significant main effects nor an interaction in a two-way repeated measures analysis of variance (rANOVA) [sentence structure (OV, SV) × subliminal stimulus (verb, NP)] (*P* > 0.09). As regards RTs, an rANOVA showed a significant main effect of sentence structure [*F*_(1, 14)_ = 5.2, *P* = 0.04] with neither a main effect of subliminal stimulus [*F*_(1, 14)_ = 0.073, *P* = 0.8] nor an interaction [*F*_(1, 14)_ = 2.7, *P* = 0.1]. A *post-hoc t*-test revealed that the RTs under the conditions with subliminal verbs were significantly greater for the SV sentences than for the OV sentences [mean difference ± s.e.m.: 41 ± 15 ms; *T*_(14)_ = 2.7, *P* = 0.02] (Figure 2B). As regards the RTs under the conditions with subliminal NPs, there was no such difference [11 ± 14 ms; *T*_(14)_ = 0.76, *P* = 0.5].

**Table 2:** **Behavioral data of the syntactic decision task performed during the MEG experiments**.

		**Subliminal stimuli**		
		**Verb**		**NP**
		**Cong**	**Incong**	
OV	Accuracy (%)	89 ± 1.4	90 ± 2.3	87 ± 2.1
	RTs (ms)	1044 ± 37	1037 ± 39	1052 ± 35
SV	Accuracy (%)	90 ± 1.8	90 ± 2.0	91 ± 2.1
	RTs (ms)	1082 ± 44	1083 ± 51	1063 ± 35

*Behavioral data (mean ± s.e.m.) of the accuracy and reaction times (RTs) are shown for each condition performed by the 15 participants. Only correct trials were included for RTs, which were measured after the target verb onset*.

We further examined the behavioral data under the conditions with subliminal verbs separately for the Cong and Incong conditions. As regards the accuracy, an rANOVA [sentence structure (OV, SV) × congruency (Cong, Incong)] showed neither significant main effects nor an interaction (*P* > 0.09). As regards the RTs, an rANOVA showed a significant main effect of sentence structure [*F*_(1, 14)_ = 7.2, *P* = 0.02] with neither a main effect of congruency [*F*_(1, 14)_ = 0.25, *P* = 0.6] nor an interaction [*F*_(1, 14)_ = 0.19, *P* = 0.7]. The increased RTs for the SV sentences irrespective of the Cong and Incong conditions suggest that subliminal verbs interfered only with the SV sentences (see the Introduction). The significant differences of RTs under the conditions with subliminal verbs, but not under the conditions with subliminal NPs, confirmed the effect of subliminal verbs irrespective of their relevance to task demands. Another possibility is that the shorter RTs under the OV sentence conditions reflect facilitatory effects of the subliminal verbs.

### Selective changes in cortical responses under the OV-Cong condition

We first checked the presence of MEG signals reflecting early visual responses to the stimuli. Such M100 and M200 components were detected both after the NP onset (Figure 2C) and after the target verb onset (Figure 2D). Figure 2E shows MEG topographies observed after the target verb onset under the OV-Cong condition. These specific temporal bins are those when significant responses were observed in the results presented below; note that the three components were outside the M200 components (see Figure 2D). A strong signal at the left frontal region was evident from the MEG topography at as early as 110–140 and 140–160 ms (Figures 2Ea,b, lateral scalp surface; the midpoint between the strongest source-sink pair). Some signals were also observed at the medial regions (Figure 2E, upper scalp surface).

**Figure 3:**
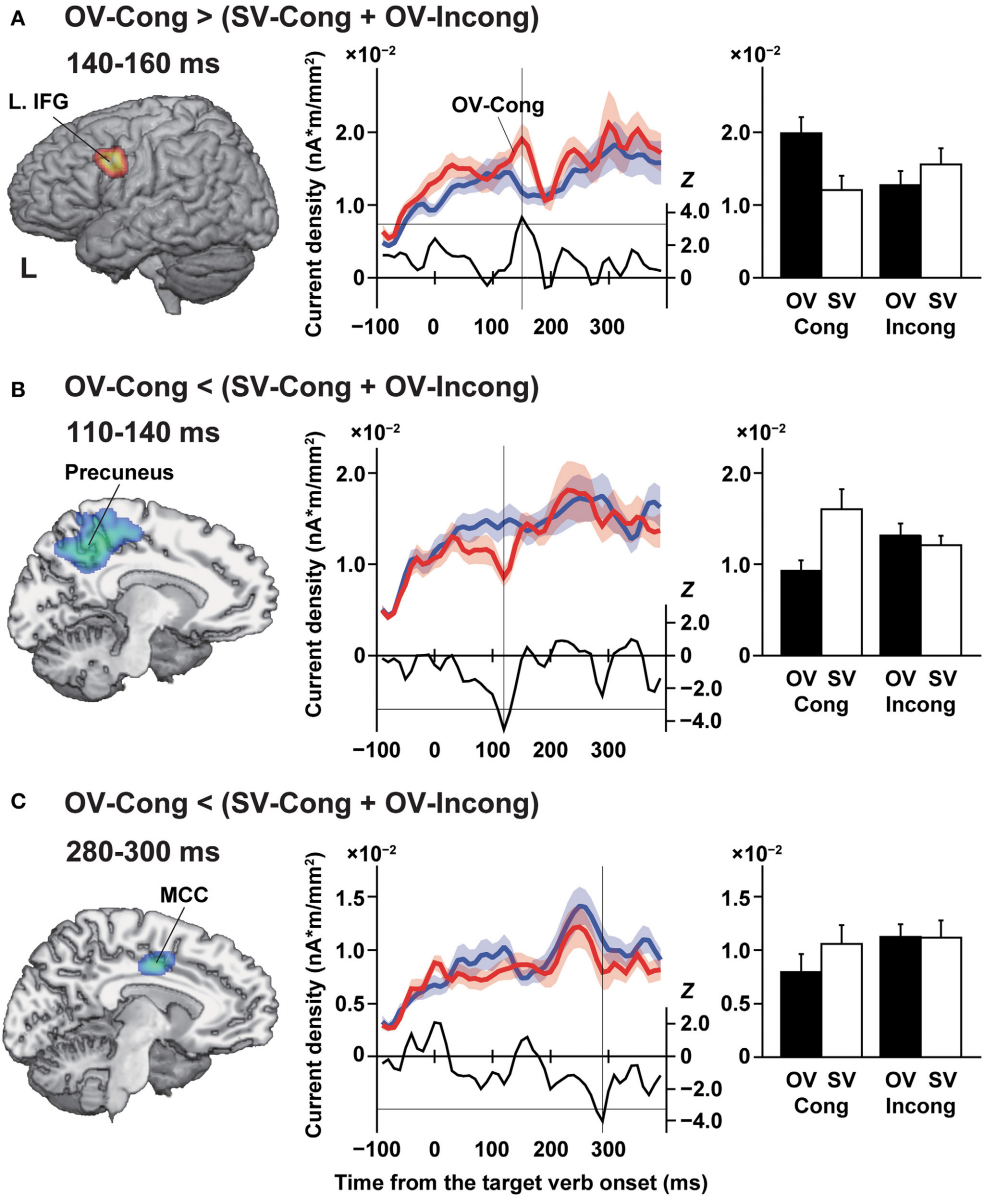
**Selective changes in cortical responses under the OV-Cong condition**. The left panels show *t*-maps on the transformed standard brain (*P*_corr_ = 0.05, false discovery rate). The middle panels show temporal changes of the current density, averaged within each significant cluster. The red and blue line graphs show the current density for the OV-Cong and (SV-Cong + OV-Incong) conditions, respectively (mean ± s.e.m., *n* = 15). The black line graphs plotted for each temporal bin show temporal changes in the *Z* values of these comparisons [positive for OV-Cong > (SV-Cong + OV-Incong)]. The horizontal black lines at *Z* = ± 3.3 denote the selection criteria of patches (uncorrected *P* = 0.001, paired *t*-test), and the vertical black lines denote temporal bins, when significant responses were observed (e.g., 150 ms for a bin of 140–160 ms). The right panels show histograms for the current density under each of four conditions (i.e., OV-Cong, SV-Cong, OV-Incong, and SV-Incong). **(A)** Significantly increased responses under the OV-Cong condition observed in the left inferior frontal gyrus (L. IFG) at 140–160 ms. The left lateral side is shown. **(B)** Significantly reduced responses under the OV-Cong condition observed in the precuneus at 110–140 ms. A para-sagittal section at *x* = −10 is shown. **(C)** Significantly reduced responses under the OV-Cong condition observed in the midcingulate cortex (MCC) at 280–300 ms. A para-sagittal section at *x* = −6 is shown.

We estimated the current density of every cortical patch under each of the four conditions, and compared OV-Cong vs. (SV-Cong + OV-Incong). Significantly increased responses to the OV-Cong condition were found in the left IFG [Talairach coordinates of the peak patch, (*x*, *y*, *z*) = (−50, 5, 29), Brodmann's areas (BAs) 44/45/6, *P*_corr_ = 0.04] at 140–160 ms (Figure 3A, left panel). According to the temporal changes in the *Z*-values of this comparison [positive for OV-Cong > (SV-Cong + OV-Incong)], the difference started to appear as early as 120 ms (Figure 3A, middle panel), which matched with the latency reported in our previous study (Iijima et al., 2009). An rANOVA [sentence structure (OV, SV) × congruency (Cong, Incong)] on the current density at 140–160 ms confirmed that there was a significant interaction of sentence structure by congruency [*F*_(1, 14)_ = 15, *P* = 0.002], with no significant main effects of sentence structure [*F*_(1, 14)_ = 2.1, *P* = 0.2] or congruency [*F*_(1, 14)_ = 0.64, *P* = 0.4] (Figure 3A, right panel). In addition, there was no significant difference in the current density between the OV-Incong condition and the OV condition with subliminal NPs [*T*_(14)_ = −1.3, *P* = 0.2], consistent with our assumption that the OV-Incong condition is ineffective and neutral regarding the enhancement.

In contrast, we observed significantly *reduced* responses under the OV-Cong condition [i.e., OV-Cong < (SV-Cong + OV-Incong)] in the precuneus [(−10, −46, 54), BA 7/31, *P*_corr_ = 0.01] at 110–130 ms (Figure 3B, left panel), extending to the superior parietal region [(−15, −54, 62), BA 7, *P*_corr_ = 0.04]. At 120–140 ms, we also observed similar responses in the superior parietal region [(−16, −37, 63), BA 7, *P*_corr_ = 0.04]. In these clusters, an immediately earlier bin (100–120 ms) also satisfied the selection criteria of patches (*Z* < −3.3) (Figure 3B, middle panel). An rANOVA on the current density at 110–140 ms showed a significant main effect of sentence structure [*F*_(1, 14)_ = 7.3, *P* = 0.02] and an interaction of sentence structure by congruency [*F*_(1, 14)_ = 11, *P* = 0.005] with no main effect of congruency [*F*_(1, 14)_ = 0.44, *P* > 0.9] (Figure 3B, right panel). This sentence structure effect is consistent with longer RTs, i.e., a larger load, for the SV sentences.

We also observed significantly reduced responses to the OV sentences in the MCC [(−6, −7, 36), BA 24, *P*_corr_ = 0.05] at 280–300 ms (Figure 3C, left panel). This temporal bin and an immediately earlier bin (270–290 ms) satisfied the selection criteria of patches (*Z* < −3.3) (Figure 3C, middle panel). An rANOVA on the current density at 280–300 ms showed a significant interaction of sentence structure by congruency [*F*_(1, 14)_ = 4.4, *P* = 0.05], with no significant main effects of sentence structure [*F*_(1, 14)_ = 2.2, *P* = 0.2] or congruency [*F*_(1, 14)_ = 3.3, *P* = 0.09] (Figure 3C, right panel). The precuneus and MCC responses were optimal solutions located in the medial wall of the brain. Based on simulated data with minimum norm estimates, the peak of estimated currents was shown to be the true deep source in the medial plane, even when the deep sources tended to be estimated in widespread regions (Hauk, 2004).

### Selective changes in partial granger causalities under the OV-Cong condition

**Figure 4:**
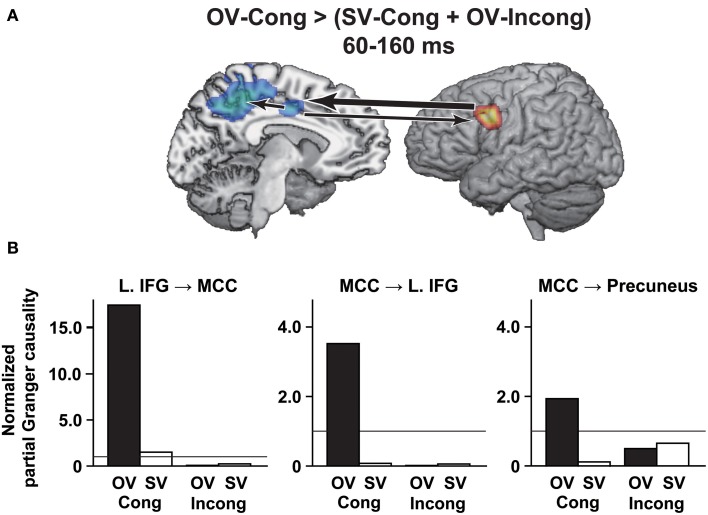
**Selective changes in partial Granger causalities under the OV-Cong condition**. Significant differences in causalities (*P*_corr_ = 0.05, false discovery rate) are schematically shown with arrows between clusters on the transformed standard brain. **(A)** A significant difference of OV-Cong > (SV-Cong + OV-Incong) in causalities at 60–160 ms, observed between the L. IFG and MCC, as well as from the MCC to the precuneus. A thick arrow denotes the strongest causality among these regions. **(B)** Histograms for the normalized partial Granger causalities shown in **(A)**. Horizontal lines at 1.0 in the histograms denote the significance of differences from zero in the normalized partial Granger causalities (*P*_corr_ = 0.05, false discovery rate).

By using the partial Granger causality analyses, we examined causal influences among the left IFG, MCC, and precuneus that showed significant responses under the OV-Cong condition in the whole-brain analyses. Between the left IFG and MCC, we found a significant difference of OV-Cong > (SV-Cong + OV-Incong) in the causalities at 60–160 ms (left IFG → MCC: *P*_corr_ < 0.002; left IFG ← MCC: *P*_corr_ < 0.002) (Figure 4A). Under the SV-Cong condition, the normalized partial Granger causality was significant, but much weaker, from the left IFG to the MCC (Figure 4B). Between the left IFG and MCC, the difference of *directed* influences, i.e., (left IFG → MCC) vs. (left IFG ← MCC), was also significant under the OV-Cong condition (*P*_corr_ < 0.002). From the MCC to the precuneus, we observed a significant difference of OV-Cong > (SV-Cong + OV-Incong) (*P*_corr_ = 0.003). Between the MCC and precuneus, the difference of directed influences was also significant under the OV-Cong condition (*P*_corr_ < 0.002). At 160–260 and 260–360 ms, there was no such significant causality (*P*_corr_ > 0.05).

## Discussion

We obtained the following results in this study. In the comparison of OV-Cong vs. (SV-Cong + OV-Incong), in which the stimulus presentation was physically controlled, we found significantly increased left IFG responses at 140–160 ms after the target verb onset (Figure 3A), confirming the existence of subliminal syntactic processes. In contrast, the precuneus and MCC responses were significantly reduced under the OV-Cong condition at 110–140 and 280–300 ms, respectively (Figures 3B,C). Finally, by means of the partial Granger causality analyses under the OV-Cong condition, we revealed a bidirectional interaction between the left IFG and MCC at 60–160 ms, as well as a significant influence from the MCC to the precuneus (Figure 4). These results indicate that a top-down influence from the left IFG to the MCC, and then to the precuneus, is critical in syntactic decisions.

Under the OV-Cong condition, we observed increased responses in the left IFG, rather than reduced responses. Recent computational theories, called “predictive coding,” have proposed that perceptual processes involve both top-down predictions and bottom-up prediction errors (Rao and Ballard, 1999; Friston, 2005; Feldman and Friston, 2010). According to these theories, two distinct layers of neurons are proposed: representational neurons and error neurons. Violation of prediction would lead to increased responses of the error neurons, while prediction causes their suppression; this relationship resembles classical dishabituation and habituation. If these two layers are assumed in all regions that are functionally equivalent in an entire brain, this assumption may not be compatible with our results, since the left IFG showed increased responses whereas the precuneus/MCC showed reduced responses. However, such regional differences could be explained by any changes in relative contribution of the two layers, depending on the hierarchical levels of each region. Moreover, “prediction” or prior information would increase responses of representational neurons by definition. Indeed, it has been proposed that the higher regions provide top-down prediction, while error neurons in the primary cortex receive bottom-up sensory inputs, separating the relative functional roles of anterior and posterior regions (Summerfield and Egner, 2009). A previous fMRI study has demonstrated that the coincidence between predicted and observed stimuli *increased* responses in the orbital prefrontal regions, suggesting the reinforcement of prior expectations (Summerfield and Koechlin, 2008). A recent MEG study has also reported that predicted words increased left IFG responses at around 150 ms (Sohoglu et al., 2012). These two studies have also indicated that prediction generated by a preceding *supraliminal* stimulus, just like our NP-type effects, remains effective for more than 1 s. Our results of the enhanced left IFG responses to congruent target verbs are consistent with the *reinforcement* of prior expectations in these predictive coding theories and previous neuroimaging studies, and further indicate that the left IFG subserves predictive syntactic processing.

Our previous MEG study showed increased left IFG responses at 120–140 ms after the verb onset of the OV sentences, indicating predictive effects during syntactic processing (Iijima et al., 2009). In the present study, we observed the left IFG responses at 140–160 ms after the target verb onset, indicating more enhanced predictive effects in OV sentences. The predictive effects thus occurred very fast as soon as a target verb appeared. The current results further showed that subliminal verbs under the OV-Cong condition indeed enhanced the left IFG responses, indicating that the predictive effects were unconscious. The predictive effects were also obligatory, since they were elicited by a preceding object, i.e., in a stimulus-driven manner; note also that the effects were elicited independently of both the transitivity of target verbs and the grammaticality of sentences (normal or anomalous); that is, they were elicited in a goal-independent manner. In general, fast, unconscious, and obligatory features support the automaticity of certain processes (Moors and De Houwer, 2006), which can thus be applied to the predictive effects.

Our previous transcranial magnetic stimulation (TMS) study showed that event-related TMS pulses facilitated syntactic decisions for OV sentences in a selective manner—i.e., only when the TMS pulses to the left IFG were administrated at 150 ms after the verb onset; this timing was also at 150 ms after the *offset* of the preceding NP (Sakai et al., 2002). It is possible that the TMS pulses temporarily raised the overall excitability of neurons, thereby creating a “stand-by” state in the left IFG, which leads to more effective activation when specific responses of those cells are required for syntactic decisions (Sakai et al., 2003). This timing is consistent with that of our present study, in which subliminal verbs were presented at 100–200 ms after the offset of the preceding NP (see Figure 1B). These results suggest that the automatic predictive effects in the left IFG were closely related to the prior state of this region.

In the precuneus, we observed significantly reduced responses at 110–140 ms under the OV-Cong condition, together with the top-down influence from the MCC at 60–160 ms. It has been reported that subliminal phonological priming reduced the precuneus responses during visual word recognition (Wilson et al., 2011), and it was also suggested that the precuneus was activated for correct responses to a target stimulus, which was incongruent with a prior stimulus (Fassbender et al., 2006). These previous results were consistent with the present ones under the conditions other than OV-Cong, suggesting that unexpected stimuli were detected at this early time range in the precuneus. The reduced precuneus responses under the OV-Cong condition are thus consistent with the suppression of error-neuron responses in posterior regions. The results of the partial Granger causality analyses indicate that the prior syntactic prediction would be transmitted from the left IFG to the precuneus through the MCC in a top-down manner, providing predictions about stimulus-specific information, such as the transitivity of a verb. After the suppression of error-neuron responses at 110–140 ms, the enhanced responses of the left IFG at 140–160 ms may reflect the reinforcement of prior expectations.

Under the OV-Cong condition, we also observed reduced MCC responses at 280–300 ms, together with a bidirectional interaction between the left IFG and MCC at 60–160 ms. Previous studies have suggested that the medial prefrontal regions, including the MCC and adjacent dorsal anterior cingulate cortex, are involved in task-set formation (Dosenbach et al., 2006; Hyafil et al., 2009). In these studies, task sets were defined as “context-appropriate stimulus-response relationships.” In the present study, the task sets for syntactic decisions were the relationships between the transitivity of the target verb and the grammaticality of the sentence (see the Introduction). Under the OV-Cong condition, task-set formation would be facilitated, since the transitivity of subliminal verbs could already specify the task sets for final responses. Such facilitation would be realized by the bidirectional interaction between the left IFG and MCC. This period includes that of increased left IFG responses at 140–160 ms, which is consistent with the involvement of the left IFG at this early time range. As a result of the established task-set formation for syntactic decisions, the contribution of the MCC would thus later be reduced at 280–300 ms under the OV-Cong condition. Under the SV-Cong condition, in contrast, the normalized partial Granger causality was significant from the left IFG to the MCC at 60–160 ms, suggesting task-set formation even under this less specified condition.

To conclude, the present study indicates that the subliminal enhancement of predictive effects is related to the generation of task sets for syntactic decisions. The elucidation of this process highlights the dynamic interactions among the identified regions, such that the MCC shares its task-set information with the left IFG to achieve automatic and predictive processes of syntax.

### Conflict of interest statement

The authors declare that the research was conducted in the absence of any commercial or financial relationships that could be construed as a potential conflict of interest.
